# The Interplay Between Autoimmune Disorders Affecting the Coagulation and Platelet Systems and Their Implications for Cardiovascular Diseases: A Systematic Review

**DOI:** 10.3390/cells14131023

**Published:** 2025-07-04

**Authors:** Kiana Mohammadian, Melika Asayesh, Fatemeh Fakhar, Shayan Keramat, Agata Stanek

**Affiliations:** 1 Division of Hematology and Blood Banking, Department of Medical Laboratory Sciences, School of Paramedical Sciences, Shiraz University of Medical Sciences, Shiraz 71348, Iran; kiana.mohammadian78@gmail.com (K.M.); fatemehfakhar1999@gmail.com (F.F.); 2 Student Research Committee, Hamedan University of Medical Science, Hamedan 65319, Iran; meli.asa0898@gmail.com; 3 VAS-European Independent Foundation in Angiology/Vascular Medicine, Via GB Grassi 74, 20157 Milan, Italy; shayan.sk1993@gmail.com; 4 Support Association of Patients of Buerger’s Disease, Buerger’s Disease NGO, Mashhad 9183785195, Iran; 5 Department of Internal Medicine, Metabolic Diseases and Angiology, Faculty of Health Sciences in Katowice, Medical University of Silesia, Ziołowa 45/47, 40-635 Katowice, Poland

**Keywords:** autoimmune diseases, coagulation disorders, platelet dysfunction, cardiovascular disease, thromboinflammation

## Abstract

Autoimmune diseases (AIDs) are chronic, heterogeneous conditions developing from an aberrant immune response, impacting particular organs or multiple systems. This systematic review attempted to investigate and evaluate the correlation between autoimmune diseases and cardiovascular disease (CVD), emphasizing immunological and pathophysiological mechanisms. A comprehensive search for relevant research was conducted on the PubMed, SCOPUS, and ScienceDirect databases, resulting in the identification of 28 studies that met the inclusion criteria. Of the cohort studies, 26 (92.8%) demonstrated a significant association between autoimmune diseases and increased cardiovascular risk. The major mechanisms include chronic inflammation, endothelial dysfunction, oxidative stress, and immune cell dysregulation. Essential biological components, including T cells, B cells, and neutrophils, were identified as contributors to atherosclerotic processes through cytokine secretion, expression of adhesion molecules, and thrombogenic activity. In contrast, two studies (7.1%) found no statistically significant association. In conclusion, autoimmune diseases significantly increase cardiovascular risk through complicated immunological mechanisms. Comprehending these pathways could influence future therapeutic approaches to reduce cardiovascular complications in affected patients.

## 1. Introduction

The coagulation system is crucial for preserving hemostasis and promoting wound healing through a precisely organized cascade of clotting factors and platelets. This system must be precisely managed to prevent improper clot formation, or thrombosis [1]. Inflammatory systemic disorders, particularly autoimmune diseases such as systemic lupus erythematosus (SLE), rheumatoid arthritis (RA), and inflammatory bowel diseases (IBDs), frequently induce aberrant activation of immunological and coagulation pathways [2]. Such interactions may result in platelet dysfunction, increased thrombotic risk, and vascular irregularities [3].

Autoimmune diseases, characterized by compromised immune tolerance and chronic inflammation, are increasingly associated with hemostatic disturbances and an elevated incidence of cardiovascular disease (CVD) [3,4]. Numerous studies demonstrate that autoimmune disorders facilitate thrombotic events through immune-mediated endothelial damage, complement activation, and procoagulant autoantibodies. Consequently, patients with autoimmune diseases face heightened risks of atherosclerosis, myocardial infarction, and various cardiovascular complications. Recognizing the relationship between immune dysregulation and coagulation dysfunction is essential for establishing innovative therapeutic approaches to mitigate cardiovascular risk in this population [2,3].

Disruptions in coagulation and platelet function significantly contribute to the development and progression of CVD [1]. In autoimmune disorders, immune-mediated endothelial damage, platelet activation, and prothrombotic conditions contribute to vascular injury and thrombotic complications [5,6]. A comprehensive understanding of the interaction between autoimmune-induced coagulation irregularities and vascular dysfunction could provide critical insights for targeted therapeutic approaches. Furthermore, the identification of biomarkers associated with autoimmune disease activity may assist in stratifying patients according to their cardiovascular risk and providing more personalized interventions [1,3,7].

The aim of this study is to summarize the critical role of the interplay between thrombotic events and autoimmunity, with a particular focus on how these mechanisms contribute to the development and progression of CVD. By analyzing existing clinical and mechanistic evidence, this review highlights underappreciated pathways and evaluates their implications for patient outcomes.

## 2. Materials and Methods

### 2.1. Literature Search

This systematic review conformed to the requirements established by the Preferred Reporting Items for Systematic Reviews and Meta-Analyses (PRISMA) guidelines. The systematic review protocol was registered in the PROSPERO database with the registration ID CRD420251032214, and a comprehensive exploration of the PubMed, SCOPUS, and ScienceDirect databases was conducted. The search terms included, but were not limited to, “autoimmune diseases,” “cardiovascular events,” “SLE,” “RA,” “ITP,” “APS,” “vascular damage,” “thrombosis,” and “atherosclerosis.” This systematic search strategy was designed to capture a broad range of studies examining the relationship between immune-mediated vascular damage, thrombosis, and accelerated atherosclerosis in autoimmune diseases. The full details of the search strategy are outlined in Table 1.

### 2.2. Study Selection

Table 2 describes the inclusion and exclusion criteria for this review. Only studies that fulfilled all specified inclusion criteria were considered eligible for review. To achieve the mentioned goal or owing to the elaborated fact, the initial database search using the predefined strategy was conducted by author K.M. Subsequently, the identified records were double-checked by author F.F. to ensure consistency with the inclusion criteria. In cases where eligibility was unclear or disagreements arose, author M.A. was responsible for resolving the conflicts through further assessment and discussion. Consensus was reached through discussion and evaluation among the main reviewers. Additionally, authors S.K. and A.S. acted as senior guarantors of the review process, overseeing the search strategy and verifying the accuracy of the inclusion process from a subject-matter perspective.

## 3. Data Extraction and Quality Assessment

The reviewers independently extracted and documented data, encompassing the author and publication year, study design, sample size, participant demographics (age, gender distribution), type of autoimmune disease, cardiovascular outcomes, and mechanisms influencing coagulation and platelets. The studies were selected based on rigorous study designs, guaranteeing suitable sampling methods and sufficient sample sizes in relation to the prevalence of autoimmune disorders. The outcome evaluation conformed to recognized clinical and epidemiological standards, emphasizing cardiovascular risk factors, inflammatory mechanisms, and thrombotic occurrences. The analysis was conducted objectively, employing statistical reporting that encompassed confidence ranges and detailed descriptions of study populations. The quality assessment verified that the chosen studies adhered to established methodological criteria, facilitating an accurate synthesis of findings and legitimate conclusions regarding the association between autoimmune disorders and cardiovascular risks.

## 4. Results

### 4.1. Search Results

**Figure 1:**
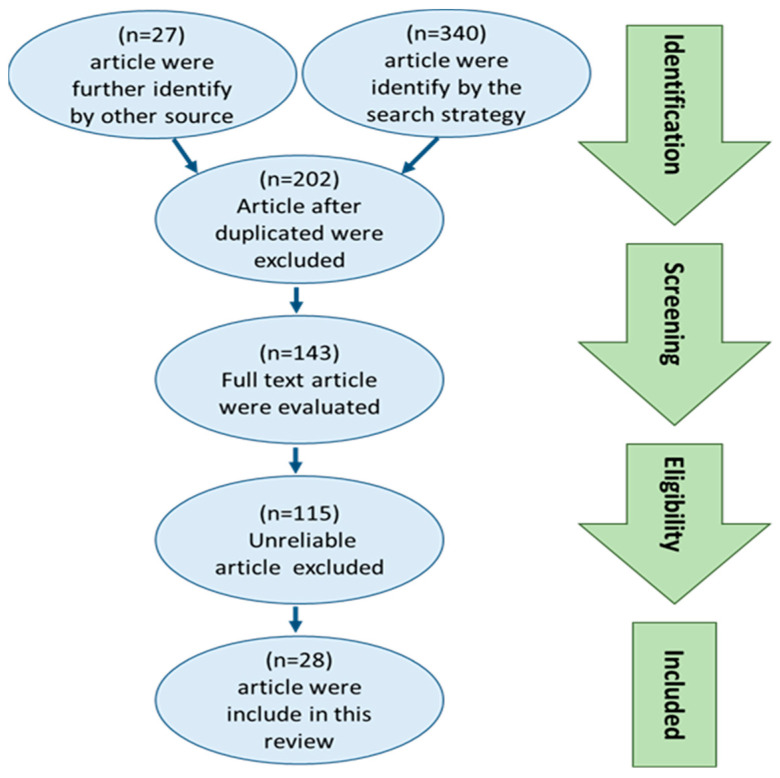
PRISMA flow diagram showing the study selection and identification. In total, 340 articles were identified, and by searching through the reference lists of relevant reviews, another 27 publications were found. In total, 202 publications were chosen after omission of duplicates, due to their title and abstract eligibility, and out of the 143 articles whose full texts were reviewed, 115 were excluded owing to faulty data. Ultimately, 28 articles were included in this review.

In Figure 1, a succinct outline is presented on the methodology of selecting the related articles. Throughout this search strategy, a total of 340 articles were found. Moreover, 27 extra publications were identified through reviewing the reference lists of the related articles. Subsequently, upon duplicates exclusion, 202 articles were chosen for eligibility evaluation based on their title and abstracts. A total of 115 articles were excluded out of 143 articles evaluated using their full texts, due to various factors such as full-text inaccessibility, patient populations, untrustworthy study designs (including case reports, ethnographies, and observational designs), or a lack of direct relevance between the two conditions. Ultimately, 28 articles were included in this review.

### 4.2. Study Characteristics

Among the 28 studies included in this systematic review, 26 (92.8%) indicated a substantial correlation between autoimmune diseases and elevated cardiovascular risk, emphasizing mechanisms including chronic inflammation, autoantibody-mediated endothelial damage, and platelet dysfunction. Conversely, two studies (7.1%) identified no statistically significant correlation.

With regard to study design, 26 (92.8%) were cohort studies, 1 (3.5%) were case–control studies, and 1 (3.5%) was a double-control study. The majority of research (25 out of 28, or 89.2%) incorporated both male and female participants, while 3 studies (10.7%) focused mainly on female patients, indicating the greater prevalence of autoimmune disorders among women.

Regarding specific autoimmune diseases, five studies (17.8%) concentrated on SLE, three studies (10.7%) focused on RA, and five studies (17.8%) investigated ITP and TTP. The subsequent investigations investigated disorders including APS, pSS, and AAV.

The findings, encompassing study design, sample characteristics, and outcomes, are presented in Table 3.

## 5. Discussion

This systematic study indicates a substantial correlation between autoimmune diseases and CVD, particularly through mechanisms of chronic inflammation, endothelial dysfunction, and platelet dysregulation.

The investigated studies demonstrate a consistent association between autoimmune diseases and increased cardiovascular risk, mostly attributable to mechanisms such as chronic inflammation, endothelial dysfunction, and platelet dysregulation. However, differences exist in the extent of cardiovascular involvement among various autoimmune disorders. The majority of research (92.8%) indicated a strong correlation between autoimmune diseases and CVD, but a small portion (7.1%) revealed no statistically significant association.

A principal feature of autoimmune diseases is chronic inflammation, which may result in endothelial dysfunction and then leads to the progression of atherosclerosis that eventually affects coagulation and platelet systems [36]. Inflammatory cytokines, including IL-1, IL-6, and TNF-α, increase the production of adhesion molecules in endothelial cells, resulting in increased platelet aggregation and the recruitment of inflammatory cells in the affected vessels [37]. Furthermore, sex-related differences in autoimmune diseases are well established, with a higher prevalence among women. Diseases such as SLE, pSS, Graves’ disease, RA, and MS demonstrate pronounced female dominance, with reported female-to-male ratios ranging from 1.7:1 to 8.8:1 (1). These disparities may be explained by differences in sex hormones, genetic susceptibility, environmental exposures, and epigenetic mechanisms (2). Particularly, although autoimmune diseases are more prevalent in females, cardiovascular complications tend to manifest with greater severity in male patients, including those with SLE and RA. Additionally, non-organ-specific autoimmunity has been detected with greater prevalence in patients exhibiting Klinefelter syndrome (47, XXY karyotype). These individuals demonstrate an elevated prevalence of antinuclear antibodies (ANAs), and certain autoimmune symptoms, such as lupus, may present with increased severity (5). This emphasizes the significance of accounting for sex chromosomal abnormalities when evaluating autoimmune-mediated cardiovascular risk.

**Figure 2:**
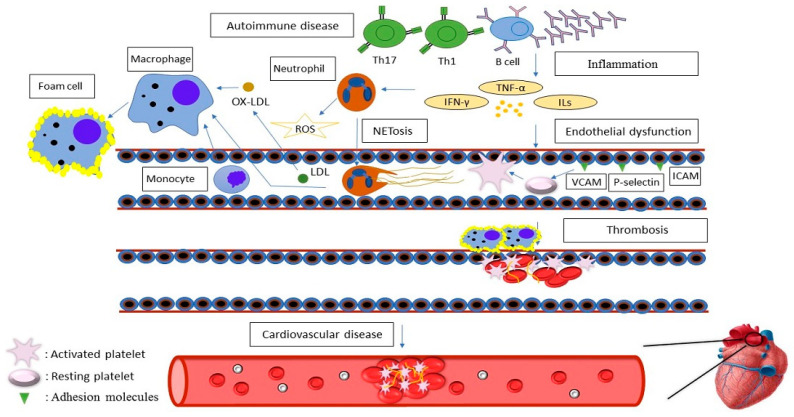
Inflammatory cytokines resulting in endothelial dysfunction. This dysfunction triggers the expression of adhesion molecules on endothelial cell surfaces, facilitating the recruitment of inflammatory cells, including platelets. Inflammation also increases neutrophils. Activated neutrophils can produce reactive oxygen species (ROS) and stimulate the migration of monocytes and other inflammatory cells, and also stimulate the activation of macrophages through NETosis. High levels of ROS contribute to the oxidation of low-density lipoprotein (LDL), which in turn attracts monocytes and transforms them into foam cells. The aforementioned factors, along with platelet activation and the subsequent initiation of the coagulation cascade, lead to clot formation and thrombosis within the vessels, ultimately resulting in cardiovascular diseases. IL, interleukin; IFN-γ, interferon gamma; Th, T helper; TNF-α, tumor necrosis factor alpha; VCAM, vascular cell adhesion; ICAM, intercellular adhesion molecule.

Endothelial dysfunction is a crucial molecular pathway in autoimmune diseases and significantly contributes to atherosclerosis and CVD (Figure 2). In autoimmune disorders, the damaged endothelium expresses adhesion molecules such as P-selectin, ICAM, and VCAM, which promote the recruitment of inflammatory cells, including platelets [36,38]. The activation of these adhesion molecules facilitates the establishment of atherosclerotic plaques and subsequent development of thrombi inside the vasculature [37,39]. This pathway emphasizes the significance of endothelial activation in associating inflammation with coagulation processes. In some autoimmune diseases, including ITP, endothelial damage may arise from antigenic mimicry between endothelial cells and platelets [40]. In RA, arterial stiffness serves as an early marker of disease progression [41]. Furthermore, endothelial dysfunction is triggered by an imbalance in the availability of vasodilators, especially nitric oxide (NO), leading to compromised vasodilation and diminished flow-mediated dilation (FMD) in SLE patients in comparison to healthy controls [42,43].

Oxidative stress is another crucial factor in the progression of atherosclerosis. Elevated levels of reactive oxygen species (ROS) result in the oxidation of low-density lipoprotein (LDL), an important step in the onset of inflammatory responses in the vascular wall [44,45]. Oxidized LDL stimulates endothelial cells, leading to the overexpression of adhesion molecules and chemoattractants that attract monocytes and change them into foam cells. These foam cells are a hallmark of atherosclerotic plaque formation [38,46]. Moreover, oxidative stress may intensify platelet aggregation, consequently increasing thrombotic events [37,47]. APS is characterized by the persistence of aPLs, especially anti-β2-glycoprotein I (aβ2GPI), which target β2GPI bound to anionic phospholipid surfaces. Oxidative stress induces a conformational modification in β2GPI, exposing immunogenic epitopes and triggering autoantibody production. These aβ2GPI antibodies activate endothelial cells, monocytes, and macrophages, leading to TF expression and initiation of the coagulation cascade through factor VIIa and downstream thrombin generation [48,49]. Additionally, aPLs interfere with natural anticoagulant mechanisms by inhibiting activated protein C (APC), elevating plasminogen activator inhibitor-1 (PAI-1), and suppressing tissue factor pathway inhibitor (TFPI), thus increasing thrombotic risk. The simultaneous effect of elevated procoagulant activity and decreased anticoagulant regulation leads to an increased occurrence of cardiovascular events in patients with APS [48,50]. Recent studies have demonstrated that platelets from APS patients, as well as healthy platelets exposed to aPLs, show increased expression of TF, a key initiator of the coagulation cascade [51]. This process involves IRAK phosphorylation and NF-κB activation and is reversed upon the inhibition of platelet heparanase activity [52]. Heparanase, an enzyme that degrades heparan sulfates, may be enhanced under stress situations such as sepsis, suggesting a potential second-hit mechanism contributing to thrombotic complications in APS [53,54].

Furthermore, recent research emphasizes the involvement of neutrophils in the progression of atherosclerosis. The total number of neutrophils demonstrates a positive correlation with plaque sizes, indicating a harmful influence of neutrophils on coronary artery disease (CAD) [55]. Activated neutrophils produce proteases and ROS, which can trigger endothelial cell activation and facilitate the migration of monocytes and other inflammatory cells. Additionally, neutrophils can participate in NETosis, resulting in the release of neutrophil extracellular traps (NETs) that enhance macrophage activation and the secretion of proinflammatory cytokines, including IL-1β and IL-18, thereby accelerating the progression of atherosclerosis [55,56]. Notably, in certain autoimmune diseases, there exists a particular subset of neutrophils identified as low-density granulocytes (LDGs). This subtype is more susceptible to releasing NETs with vasculopathic and immunostimulatory characteristics compared to neutrophils of normal density. In SLE, these LDGs exhibit an enhanced capacity to produce proinflammatory cytokines, particularly type I interferons [57]. Lipid rafts are essential in APS by aggregating critical receptors such as TLR4, ANXA2, and LRP8 on endothelial cells, monocytes, and platelets. Anti-β2GPI antibodies accumulate in these membrane domains, initiating proinflammatory signaling pathways, including MyD88, IRAK, and NF-κB activation. This results in the expression of TF and the release of TNF-α, promoting a procoagulant environment. The participation of lipid rafts has been confirmed by co-immunoprecipitation and raft-disrupting substances, emphasizing their significance as essential platforms for pathogenic signaling in APS [58]. Recent studies have discovered new signaling pathways involved in thrombosis in APS. Anti-β2GPI antibodies interact with LRP6 and PAR-2 in lipid rafts on endothelial cells, thereby activating β-catenin and promoting TF production [59,60]. Inhibiting this pathway, such as with DKK1, diminishes procoagulant reactions. Furthermore, the activation of mTORC1/2 by aPLs in endothelial cells is associated with vascular remodeling, whereas the EPCR–LBPA–TLR7 axis facilitates B1a cell activation and the prolonged production of aPLs. These mechanisms illustrate how aPLs induce thrombosis through immune–coagulation interactions, providing novel therapeutic targets beyond anticoagulation [58,61].

T cells also play a crucial role in atherosclerosis. Specifically, Th1 and Th17 cells, activated during immune responses, specifically contribute to plaque development and instability in atherosclerosis. Th1 cells, by secreting IFN-γ and TNF-α, stimulate inflammatory responses that increase plaque instability and exacerbate cardiovascular diseases [62,63]. On the other hand, Th17 cells that release IL-17 increase the expression of adhesion molecules and attract inflammatory cells to areas of vascular damage, thus aggravating atherosclerotic lesions [64,65]. The difference between Th17 and regulatory T cells (Tregs) in autoimmune disorders, including RA, MS, and SLE, enhances inflammation and accelerates the risk of atherosclerosis and CVD [66]. For instance, in SLE, the proliferation of Th17 cells is associated with atherosclerosis and disease activity, but Tregs are diminished in SLE-related CVD. The metabolic characteristics of SLE T cells, such as mitochondrial malfunction, glutaminolysis, increased glycolysis, active lipid synthesis, and heightened mTOR activation, are the fundamental reasons for the Th17/Treg cell imbalance in SLE patients [67].

B lymphocytes also play a role in the pathophysiology of atherosclerosis and cardiovascular disorders. B1 and follicular B cells contribute to the formation of atherosclerotic plaques by producing autoantibodies and cytokines [68,69,70]. In autoimmune diseases, including lupus, IgG autoantibodies can activate endothelial cells and enhance platelet aggregation, consequently worsening atherosclerosis, but IgM antibodies may have a protective function [68,71].

SLE and RA exhibit fundamental immunoinflammatory mechanisms that are common with APS, such as persistent endothelial dysfunction, immune complex accumulation, and persistent cytokine secretion. In SLE, antiphospholipid antibodies exacerbate vascular damage by diminishing nitric oxide production, increasing oxidative stress, and activating complement, thus facilitating accelerated atherosclerosis. Likewise, RA promotes a proatherogenic condition by sustained elevation of IL-6, TNF-α, and CRP, resulting in endothelial damage and heightened platelet aggregation [72,73]. These mechanisms demonstrate SLE and RA as independent cardiovascular risk factors, surpassing conventional scoring methods [73].

In summary, increasing our understanding of antithrombotic treatments across autoimmune diseases is critical for refining clinical decision-making and enhancing patient outcomes in the face of complicated coagulation abnormalities. Recent advancements have highlighted the need for tailored antithrombotic strategies in autoimmune conditions associated with hemostatic abnormalities [74]. In vascular APS, patients with persistent aPLs are encouraged to receive lifelong anticoagulant therapy, while asymptomatic carriers benefit from prophylaxis during high-risk scenarios. Pregnancy provides a particular challenge, requiring the switch from oral anticoagulants to heparin in vascular APS cases and the administration of low-dose aspirin (LDA) and/or heparin in obstetric APS to prevent serious adverse consequences [75]. In SLE, where traditional treatment focuses on immunosuppression with hydroxychloroquine (HCQ), corticosteroids, and recently approved biological therapies such as belimumab, long-term survival has disclosed an increasing thrombotic burden. Agents such as HCQ exhibit antithrombotic properties through the inhibition of platelet aggregation and the lowering of blood viscosity, suggesting they are advantageous in both SLE and APS [76]. Furthermore, low-dose aspirin is advised for asymptomatic aPL carriers and SLE patients to mitigate thrombotic risk, as confirmed by cohort and meta-analysis studies. Warfarin continues to be the primary agent for APS prophylaxis, although its teratogenic risk restricts its application in early pregnancy [77]. Low-molecular-weight heparin (LMWH), with minimal bleeding risk and immunomodulatory features including complement inhibition, is generally recommended in pregnancy and high-risk SLE patients [76]. In the context of iTTP, caplacizumab, a nanobody targeting the vWF–platelet axis, has emerged as an effective adjunct therapy, decreasing mortality and relapse prevalence in clinical studies. However, real-world information on its safety in elderly populations remains limited, and its bleeding risk demands personalized risk assessment, especially when associated with anticoagulant medications [78].

In light of the essential role of chronic inflammation and hemostatic abnormalities in precipitating cardiovascular complications in autoimmune diseases, immediate identification and rapid intervention are crucial. The administration of medicines such as HCQ, LDA, and LMWH in high-risk patients, especially during pregnancy or in individuals with persistent aPLs, may mitigate thrombotic occurrences and vascular damage. Implementing specific treatment options and conducting cardiovascular risk assessment may significantly enhance clinical results in this susceptible population.

## 6. Conclusions

In conclusion, this systematic review provides integrative insight into how autoimmune diseases contribute to cardiovascular disease (CVD), emphasizing the underrecognized role of coagulation and platelet dysfunction as mediators beyond classical inflammatory pathways. While inflammation and endothelial activation remain critical contributors, our analysis highlights the importance of disease-specific hemostatic alterations, such as the persistence of antiphospholipid antibodies, ADAMTS13 deficiency, and complement activation, that uniquely predispose autoimmune patients to thrombotic events. These findings refine our understanding of CVD pathogenesis in autoimmunity by linking immune dysregulation with procoagulant cellular signaling (e.g., via lipid rafts, mTOR, or platelet heparanase pathways) in conditions like APS, SLE, RA, and iTTP. Importantly, we observed heterogeneity in cardiovascular outcomes across diseases, suggesting that tailored risk stratification and antithrombotic strategies are warranted rather than generalized approaches. Clinical implications extend to the judicious use of agents such as hydroxychloroquine, low-dose aspirin, and LMWH, particularly in high-risk populations such as pregnant women and elderly patients. Future research should prioritize mechanistic dissection of immune–thrombosis crosstalk, the validation of predictive biomarkers, and longitudinal trials evaluating cardiovascular outcomes in patients receiving immunomodulatory therapies. Ultimately, this evolving knowledge may guide personalized management to prevent CVD complications in autoimmunity more effectively.

## Figures and Tables

**Table 1:** Full details of the search strategy terms.

Terms ^1^	Search Strategy Terms
Term 1	“Autoimmune disease” “Inflammation” OR “Immune dysregulation” “Thrombosis” OR “Endothelial dysfunction” OR “Autoimmune disorders” OR “Autoimmunity” OR “Autoinflammation” OR “Autoantibodies”
Term 2	“Cardiovascular disease (CVD)” OR “Atherosclerosis” OR “Myocardial infarction” OR “Heart failure” OR “Peripheral arterial disease (PAD)” OR “Coronary heart disease (CHD)”
Term 3	“Oxidative stress” OR “Endothelial dysfunction” OR “Platelet dysfunction” OR “ADAMTS13 activity” OR “Complement activation” OR “Chronic inflammation”
Term 4	“Clinical Trials” OR “Cohort Study” OR “Case–Control Study” OR “Systematic Review”

^1^ Terms 1, 2, 3, and 4 were joined with “AND”.

**Table 2:** Inclusion and exclusion criteria.

Inclusion Criteria	Exclusion Criteria
Studies about the effect of autoimmune disease on the coagulation system	Animal and in vitro studies
Studies that clearly stated that autoimmune diseases contribute to the development of cardiovascular disease through their effects on the coagulation system	Studies with no connection between autoimmune disease and the coagulation system
Studies that involve both men and women or one gender alone	Studies on autoimmune diseases that affect the coagulation system but not the cardiovascular system
Studies that investigated autoimmune diseases with direct or indirect effects on the coagulation system or platelet function.	Studies lacking information about the effect of autoimmune diseases on the cardiovascular system
Studies that reported cardiovascular outcomes as a consequence of autoimmune-induced coagulation or platelet abnormalities.	Studies with a very small sample size
Studies that included an adequate sample size relevant to the disease prevalence and study design.	

**Table 3:** The summarized results of human studies that fulfilled the inclusion criteria.

Number	Study	Year	Type of Study	Sample Size	Age	Gender	Autoimmune Disease	Mechanisms Affecting Coagulation and Platelets	Cardiovascular Disease
1 [8]	Adelborg et al.	2018	cohort study	3584	≥18	Both genders: Male: 42% Female: 58%	chronic immune thrombocytopenia (cITP)	Low platelet counts have been associated with increased risks of both hemorrhage and mortality.	CVD events possibly arise due to an imbalance in hemostasis and platelet activation in chronic ITP.
2 [9]	Chandan et al.	2018	cohort study	6591	Mean: 48.4	Both genders: Male: 41.4% Female: 58.6%	ITP	Platelet microparticles (PMPs) released in ITP may increase vascular inflammation and thrombosis.	Atherosclerosis and thrombotic CVD linked to a procoagulant state mediated by PMPs.
3 [10]	Haseefa et al.	2020	cohort study	NA	NA	Both genders	ITP	Some investigators propose that autoantibodies associated with ITP may induce endothelial damage through antigenic mimicry between coronary endothelial cells and platelets.	ITP demonstrated a significant correlation with Aortic valve disease (AVD) over a decade.
4 [11]	Brodsky et al.	2021	cohort study	181	Mean: 39	Both genders: Male: 28.7% Female: 71.3%	Immune-mediated thrombotic thrombocytopenic purpura (iTTP)	iTTP causes ADAMTS13 deficiency, leading to microvascular platelet-rich thrombi.	CVD is a long-term complication due to endothelial damage from recurrent thrombotic episodes.
5 [12]	Sukumar et al.	2022	cohort study	222	Mean: 42	Both genders: Male: 29.7% Female: 70.3%	iTTP	Decreased ADAMTS13 activity contributes to microvascular platelet aggregation, thrombocytopenia, microangiopathic hemolytic anemia, and different end-organ dysfunction.	CVD occurs in survivors of iTTP relapse due to sustained microvascular thrombotic burden.
6 [13]	C. Somers et al.	2012	cohort study	95	Mean: 37.66 ± 9.1	Female: 100%	SLE	Preclinical vascular damage, decreased flow-mediated dilatation (FMD), increased carotid intima media thickness (CIMT), and coronary calcification severity lead to increased risk of SLE.	This study indicates the progression of atherosclerosis in lupus patients
7 [14]	A. Hassan et al.	2013	Case–control study	120	Mean: 32	Both genders: Male: 8.4% Female: 91.6%	SLE	Autoantibodies, immune complex formation, and autoantibody deposition significantly increase thrombotic parameters in SLE patients, including plasma fibrinogen, vWF antigen, tPA antigen, and fibrin D-dimer.	Increased prevalence of PAD in patients with SLE.
8 [15]	M. Bartels et al.	2014	cohort study	70	Mean: 52	Both genders: Male: 19% Female: 81%	SLE	Not reported. Likely chronic inflammation and platelet activation in SLE.	Elevated CVD risk despite mild SLE, possibly due to subclinical thromboinflammation.
9 [16]	Antonio Aviña-Zubieta et al.	2017	cohort study	4863	Mean: 48.9	Both genders: Male: 14% Female: 86%	SLE	Not clearly stated; implied systemic inflammation and prothrombotic autoimmunity	Increased cardiovascular events in SLE patients likely mediated by inflammatory endothelial dysfunction.
10 [17]	Pujades-Rodriguez et al.	2016	cohort study	12,120	Mean: 57	Both genders: Male: 27.7% Female: 72.3%	Rheumatoid arthritis (RA)	Not clearly stated.	Individuals diagnosed with RA demonstrate increased rates of an acute myocardial infarction (AMI), unanticipated coronary mortality, heart failure, cardiac arrest, and PAD.
11 [18]	Baragetti et al.	2018	cohort study	40	Mean: 42 + 9	Both genders: Male: 10% Female: 90%	SLE	Specific T cell subsets have been associated with the long-term development of atherosclerosis and can be helpful in predicting vascular disease progression.	SLE patients have higher carotid atherosclerosis burden and increased cardiovascular risk, exacerbated by common classical cardiovascular risk factors and systemic inflammation.
12 [19]	Chen et al.	2018	cohort study	10,568	less than 45 years	Both genders: Male: 25.95% Female: 74.05%	RA	RA provokes chronic inflammation and immune dysregulation, which subsequently impairs arterial walls through sustained endothelial dysfunction and leads to markedly elevated arterial stiffness.	Independent correlation with CVD in young adults through inflammation-induced vascular dysfunction.
13 [20]	Argnani et al.	2021	cohort study	21,201	Mean: 61.7 ± 13.7	Both genders: Male: 22% Female: 76%	RA	Not detailed.	An increased risk of cardiovascular events, including atrial fibrillation, heart failure, and myocardial infarction, as well as an elevated mortality rate compared with the general population.
14 [21]	Di Minno et al.	2019	cohort study	192	Mean: 49.84 ± 12.0	Both genders: Male: 18% Female: 82%	Antiphospholipid antibodies (aPLs)	Not detailed.	Elevated CVD incidence likely due to hypercoagulability induced by aPLs
15 [22]	Selmi et al.	2020	cohort study	1712	Mean: 47.2	Both genders: Male: 50.2% Female: 49.8%	aPL	Not detailed.	APLs correlate with subclinical atherosclerosis and an increased incidence of cardiovascular events, particularly in individuals with elevated cardiovascular risk.
16 [23]	P.R.J. AMES et al.	2009	Case double- control study	49	Mean: 37 ± 11	Both genders: Male: 36.8% Female: 63.2%	antiphospholipid syndrome (APS)	The persistence of aPL leads to thrombosis in patients with primary antiphospholipid syndrome (PAPS).	Premature atherosclerosis is a clinical characteristic for individuals with thrombotic PAPS.
17 [24]	Conti et al.	2014	cohort study	Total: 68 PAPS: 18 SLE: 50	Mean of PAPA: 39.9 ± 11.5 Mean of SLE: 40 ± 11.2	PAPS: Both genders: Male: 22.3% Female: 77.7% SLE: Both genders: Male: 12% Female: 88%	APS and SLE	The immune response to β2 glycoprotein I (β2GPI), the primary target of aPL, may link autoimmune chronic damage with endothelial dysfunction, exacerbating proinflammatory conditions, and maintaining pathogenic antibody release.	A considerable proportion of individuals with SLE and APS correlated with subclinical atherosclerosis.
18 [25]	Andrade et al.	2016	cohort study	27	≥55	Female: 100%	APS	Not detailed.	Patients with PAPS have no evidence of premature atherosclerosis and do not appear to possess an elevated risk of developing it.
19 [26]	Kravvariti et al.	2018	cohort study	Total: 86 PAPS: 43, Systemic lupus erythematosus-associated APS (SLE/APS): 43	Mean:46	PAPS: Both genders: Male: 37% Female: 63% SLE/APS: Both genders: Male: 16% Female: 84%	APS and SLE/APS	Not detailed.	Individuals with PAPS and SLE/APS have a higher risk of atherosclerotic plaques in the carotid and femoral arteries.
20 [27]	Bettiol et al.	2020	cohort study	Total: 167 thromboticAPS: 131, Obstetric APS: 36	Mean of thrombotic APS: 49.44, Mean of Obstetric APS: 51.14	thrombotic APS: Female: 100% Obstetric APS: Female: 100%	APS	Not detailed.	Thrombotic APS correlates with elevated markers of subclinical atherosclerosis.
21 [28]	Wu et al.	2018	cohort study	4175	Mean: 50.15 ± 16.80	Both genders: Male: 24.6% Female: 75.4%	Primary Sjögren’s syndrome (pSS)	Not detailed.	pSS has been associated with a heightened risk of future coronary heart disease (CHD).
22 [29]	Łuczak et al.	2021	cohort study	46	Mean: 47.34 ± 11.9	Both gender: Male: 6.6% Female: 93.4%	pSS	Anti-Ro/SSA antibodies impair Flow-mediated dilation (FMD) and contribute to endothelial dysfunction.	No apparent CVD reported; however, endothelial dysfunction suggests a potential atherogenic risk.
23 [30]	Sieiro Santos et al.	2023	cohort study	102	Mean: 65 ± 24	Both genders: Male: 18% Female: 82%	pSS	Autoantibodies and complement activation promote thrombosis and endothelial injury.	Increased coronary artery disease and venous thrombosis as outcomes of an autoimmune prothrombotic state.
24 [31]	Sun et al.	2023	cohort study	5092	Mean: 57	Both genders: Male: 12.7% Female: 87.3%	pSS	Not specified; assumed chronic immune activation and vascular involvement in pSS.	Elevated risk of heart failure and cardiovascular incidents in pSS, possibly associated with inflammation and thrombosis.
25 [32]	Aaramaa et al.	2024	cohort study	Total: 7558 Seropositive RA: 2368, Seronegative RA: 916, Ankylosing spondylitis (AS): 715 Psoriatic arthritis (PsA): 923, pSS: 412, SLE: 190, Gout: 2034	Mean of Seropositive RA: 56.2, Seronegative RA: 54.4, AS: 40.4, PsA: 51.6, pSS: 53.3, SLE: 47.5, Gout: 65.7	Seropositive RA: Both genders: Male: 30.2% Female: 69.8%, Seronegative RA: Both genders: Male: 27.1% Female: 72.9%, AS: Both genders: Male: 49.2% Female: 50.8%, PsA: Both genders: Male: 44.6% Female: 55.4%, pSS: Both genders: Male: 8% Female: 92%, SLE: Both genders: Male: 11.1% Female: 88.9%, Gout: Both genders: Male: 81.1% Female: 18.9%	Seropositive RA, Seronegative RA, AS, PsA, pSS, SLE, Gout	Not detailed.	CVD risk is highest in SLE and gout, possibly associated with autoimmune-driven coagulopathy and vascular inflammation.
26 [33]	Massicotte-Azarniouch et al.	2022	cohort study	1520	Mean: 60.8	Both gender: Male: 49.5% Female: 50.5%	Anti-neutrophil cytoplasmic antibody (ANCA)-associated vasculitis (AAV)	AAV causes necrotizing vasculitis and endothelial injury, promoting thrombogenesis.	Increased cardiovascular risk following diagnosis attributed to extensive vascular damage and thrombotic tendency.
27 [34]	Vegting et al.	2023	cohort study	144	Mean: 62	Both genders: Male: 56% Female: 44%	AAV	Not detailed.	In this study, there was a substantial association between patients with AAV and cardiovascular events that persisted.
28 [35]	Nygaard et al.	2024	cohort study	2371	Mean: 63	Both genders: Male: 53.7% Female: 46.3%	AAV	Inflammation and necrosis of blood arteries leading to thrombus development and/or narrowing of coronary vessels, and can aggravate pre-existing subclinical atherosclerosis.	CVD was observed as a downstream result of autoimmune-mediated thromboinflammation in AAV

cITP, chronic immune thrombocytopenia; ITP, immune thrombocytopenic purpura; iTTP, immune-mediated thrombotic thrombocytopenic purpura; ADAMTS13, a disintegrin and metalloproteinase with thrombospondin motifs 13; FMD, flow-mediated dilation; CIMT, carotid intima media thickness; SLE, systemic lupus erythematosus; RA, rheumatoid arthritis; aPL, antiphospholipid antibodies; APS, antiphospholipid syndrome; PAPS, primary antiphospholipid syndrome; pSS, primary Sjögren’s syndrome; AAV, ANCA-associated vasculitis; CVD, cardiovascular disease; PAD, peripheral artery disease; AMI, acute myocardial infarction; CHD, coronary heart disease; PMPs, platelet microparticles; vWF, von Willebrand factor; tPA, tissue plasminogen activator.

## Data Availability

We used PubMed, SCOPUS, and ScienceDirect databases to screen articles for this systematic review. We do not report any data.

## Abbreviations

The following abbreviations are used in this manuscript:

cITP	Chronic immune thrombocytopenia
ITP	Immune thrombocytopenic purpura
iTTP	Immune-mediated thrombotic thrombocytopenic purpura
ADAMTS13	A disintegrin and metalloproteinase with thrombospondin motifs 13
FMD	Flow-mediated dilation
CIMT	Carotid intima media thickness
SLE	Systemic lupus erythematosus
RA	Rheumatoid arthritis
aPLs	Antiphospholipid antibodies
APS	Antiphospholipid syndrome
PAPS	Primary antiphospholipid syndrome
pSS	Primary Sjögren’s syndrome
AAV	ANCA-associated vasculitis
CVD	Cardiovascular disease
PAD	Peripheral artery disease
AMI	Acute myocardial infarction
CHD	Coronary heart disease
PMPs	Platelet microparticles
vWF	von Willebrand factor
tPA	Tissue plasminogen activator
NETs	Neutrophil extracellular traps
